# Relationship between preoperative size of rotator cuff tears measured using radial-slice magnetic resonance images and postoperative rotator cuff integrity: a prospective case-control study

**DOI:** 10.1016/j.jseint.2021.11.005

**Published:** 2021-12-17

**Authors:** Yuji Shibayama, Toshiaki Hirose, Akira Sugi, Emi Mizushima, Yuto Watanabe, Rira Tomii, Kousuke Iba, Toshihiko Yamashita

**Affiliations:** a Department of Orthopaedic Surgery, Sapporo Medical University School of Medicine, Sapporo, Hokkaido, Japan; b Asabu Orthopaedic Hospital, Sapporo, Hokkaido, Japan

**Keywords:** Radial magnetic resonance imaging, Rotator cuff tear, Retear, Risk factor, Prognostic factors, Cuff integrity

## Abstract

**Background:**

Magnetic resonance imaging (MRI) is useful for diagnosing shoulder diseases preoperatively. However, preoperative risk factors for retears have not been previously reported using a radial-slice MRI. Here, we investigated the relationship between the preoperative tear area of the rotator cuff evaluated using radial-slice MRI and the postoperative rotator cuff integrity. Our hypothesis is that larger tear area of the rotator cuff measured using radial-slice MRI would be associated with increased retear rates.

**Methods:**

From June 2010 to October 2015, we treated 102 consecutive patients who underwent shoulder arthroscopy for reparable rotator cuff tears. The patient demographics, medical comorbidities, radiologic factors, tear size, fatty infiltration, muscle atrophy measured using oblique coronal and oblique sagittal MRI, and the tear area calculated using radial-slice MRI were assessed to compare the intact and retear groups in univariate and multivariate logistic regression analyses. The cutoff values of the independent factors were obtained using the receiver operating characteristic curve.

**Results:**

Retears occurred in 15 of 102 (14.7%) patients. In the univariate analysis, significant differences were found between the two groups for tear size, fatty infiltration of the supraspinatus and infraspinatus, muscle atrophy, and tear area. In the multivariate analysis, the tear area was the independent factor that significantly affected the rate of retear. A tear area of 6.3 cm^2^ was the strongest predictor of retear with an area under the curve of 0.965, sensitivity of 86.7%, and specificity of 96.6%.

**Conclusion:**

The tear area was the independent factor that most significantly affected the rate of retear and showed excellent accuracy with a cutoff value of 6.3 cm^2^. Radial-slice MRI may be a valuable diagnostic tool for assessing the postoperative rotator cuff integrity.

Rotator cuff tears are a common cause of shoulder pain and dysfunction, and the global prevalence is about 40% in people over the age of 60 years.^8^^,^^32^ In most cases, an arthroscopic rotator cuff repair provides satisfactory clinical results.^17^^,^^28^^,^^38^ However, retear of a repaired tendon sometimes occurs, and several studies have revealed that retears lead to inferior postoperative clinical outcomes.^1^^,^^4^^,^^21^^,^^34^^,^^38^ There are several perioperative factors that increase the chances of retear after repair.^22^^,^^24^^,^^33^ An increased tear size, muscle atrophy, and fatty infiltration are known to be the most important factors leading to increased retear rates.^13^^,^^39^ Therefore, it is important to evaluate the condition of the rotator cuff using preoperative magnetic resonance imaging (MRI) to reduce the rate of retear. However, very few studies indicate cutoff values, which could aid in judging the prognosis of rotator cuff tears. Kim et al reported that the cutoff values for predicting retear were extent of retraction of 22.2 mm (sensitivity of 75.0%, specificity of 77.0%) and occupation ratio of 53.5% (78.6%, 78.9%).^20^ In addition, Nozaki et al suggested that the optimal cutoff value was fat fractions in the supraspinatus muscle of 26.6% (sensitivity of 70.6%, specificity of 80.0%) and 31.0% (93.1%, 65.0%), respectively.^31^ From these results, it is desirable to consider alternate imaging of the shoulder with a higher diagnostic rate to prevent retear.

Recently, a few reports have suggested that radial-slice MRI is useful for the diagnosis of rotator cuff tears.^10^^,^^14^ Radial-slice MRI that produces cross slices perpendicular to rotator cuff insertions may efficiently capture rotator cuff tears. Particularly, radial-slice MRIs provide good visualization of the tear morphology.^34^ However, to our knowledge, the preoperative risk factors for retear using radial-slice MRI have not been previously reported. This study aimed to investigate the relationship between the preoperative tear area of the rotator cuff evaluated using radial-slice MRI and the postoperative rotator cuff integrity. Our hypothesis is that retear rate is correlated to larger tear area of the rotator cuff measured using radial-slice MRI.

## Materials and methods

### Patient selection

This study was approved by the clinical ethics committee at the authors’ hospital. Between June 2010 and October 2015, we treated 114 consecutive patients who underwent shoulder arthroscopy for rotator cuff tears by a single senior surgeon at a particular institution. We had acquired postoperative follow-up data for a minimum of 2 years with MRI having been performed at the final follow-up. The patients comprised 62 men and 40 women, and the average age of the patients was 67.0 ± 8.71 years (range, 49-87 years). The exclusion criteria were previous shoulder surgery, cuff tear arthropathy, isolated tears of subscapularis, incompletely repaired tendon, and irreparable rotator cuff tears.

### Patient evaluation

Patient charts were evaluated for age, sex, history of smoking, hand dominance, duration of symptoms, body mass index (BMI), and medical comorbidities (hypertension, hypercholesterolemia, and diabetes). This medical information is routinely documented on intake questionnaires.

### Radiographic assessment

Radiologic factors, including the acromiohumeral interval (AHI),^23^ and critical shoulder angle (CSA)^29^ were measured preoperatively using the shoulder true anteroposterior (AP) view of a simple radiograph taken preoperatively. The AHI was measured as the shortest distance between the dense cortical bone of the undersurface of the acromion and the most proximal articular cortex of the humeral head. The CSA was measured between a line connecting the superior and inferior bone margins of the glenoid and a line from the inferior bone margin of the glenoid to the most inferolateral point of the acromion.

### MRI assessment

All preoperative MRIs were performed within 1 month before the surgery. We used a 3.0-T MRI unit (Signa HDx 3.0T; GE Healthcare Bioscience, Piscataway, NJ). The tear size, fatty infiltration, and muscle atrophy were measured using oblique coronal and oblique sagittal magnetic resonance images acquired using proton density T2-weighted imaging (repetition time, 4000 milliseconds; echo time, 90 milliseconds) with a slice thickness of 3.0 mm. The AP tear length was measured in the AP dimension on oblique sagittal images, and the mediolateral (ML) tear length was measured by the straight line distance between the lateral margin of the footprint of the supraspinatus and the medial margin of the retracted cuff on oblique coronal images.^6^ Fatty infiltration of the supraspinatus, infraspinatus, and subscapularis was measured using the Goutallier classification as modified by Fuchs et al in the scapular Y-view using an oblique sagittal slice.^9^ Fatty infiltration was classified into 5 stages scored from 0 to 4: stage 0, normal; stage 1, some fatty streaks; stage 2, fatty infiltration throughout with less fat than muscle; stage 3, fatty infiltration with equal muscle and fat; and stage 4, fatty infiltration with more fat than muscle.^9^^,^^12^

Muscle atrophy was quantified by measuring the occupation ratio of the muscle belly in the supraspinatus fossa using the method of Thomazeau et al.^40^ Muscle atrophy of the supraspinatus muscle was also evaluated by using the tangent sign, which is considered negative when the superior border of the supraspinatus is superior to the line tangential to the coracoid and scapular spine.^25^

**Figure 1:**
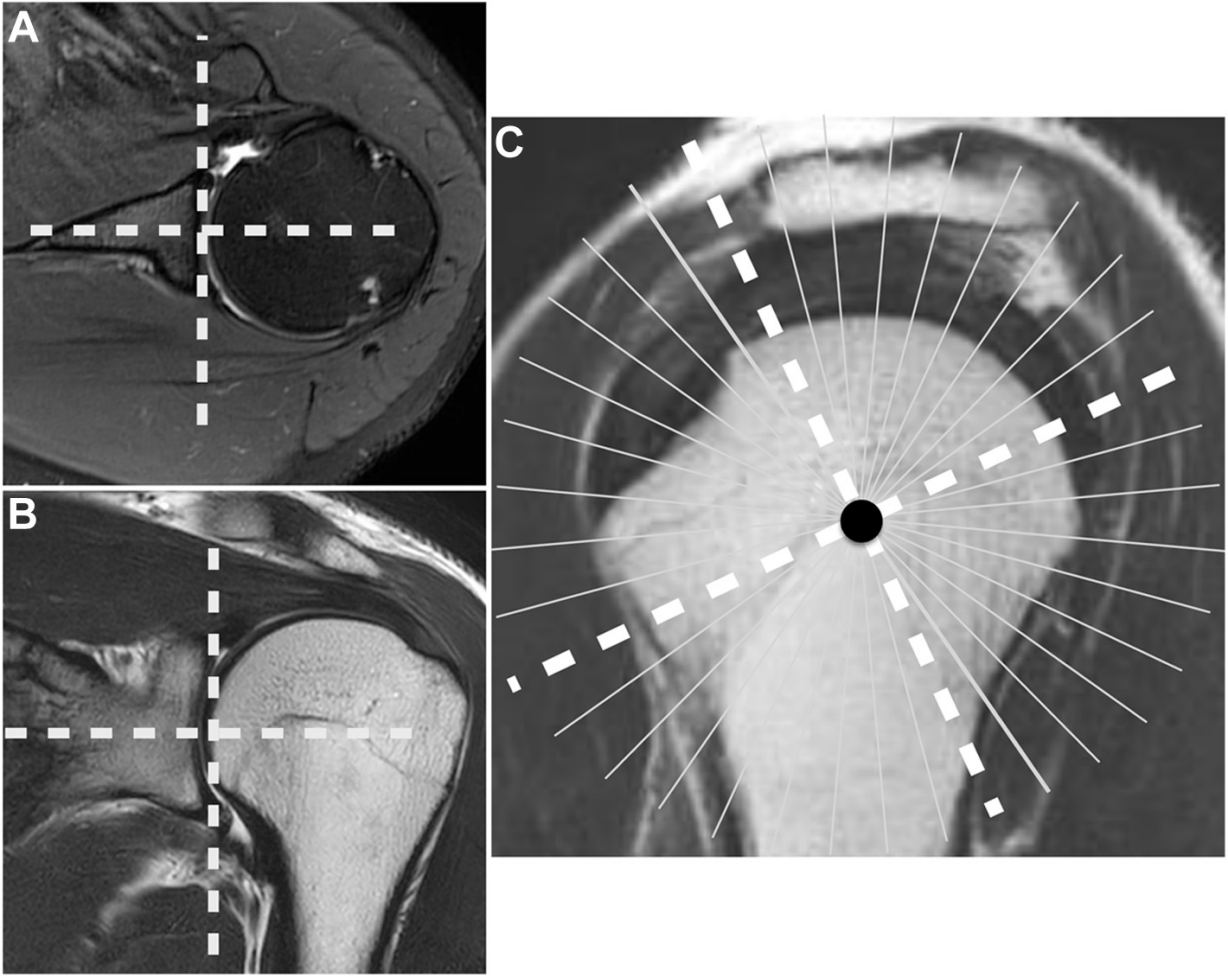
Imaging procedure used for radial slices. First, the rotation axis (*dashed line*) was defined by using (**A**) axial and (**B**) coronal scans. (**C**) Second, this axis was used to obtain 18 slices (*white line*) per 10° rotational angles.

The tear area of the rotator cuff was measured using radial-slice MRI scans acquired using fat-suppressed T2-weighted imaging (repetition time, 6000 milliseconds; echo time, 60 milliseconds) with a slice thickness of 3.0 mm. The imaging procedure for the radial slices inluded the following steps: First, the rotation axis was defined as the line passing through the center of the humeral head and the center of the glenoid by using axial and coronal scans; second, according to the axis, 18 slices were obtained per 10° rotationally (Fig. 1).

**Figure 2:**
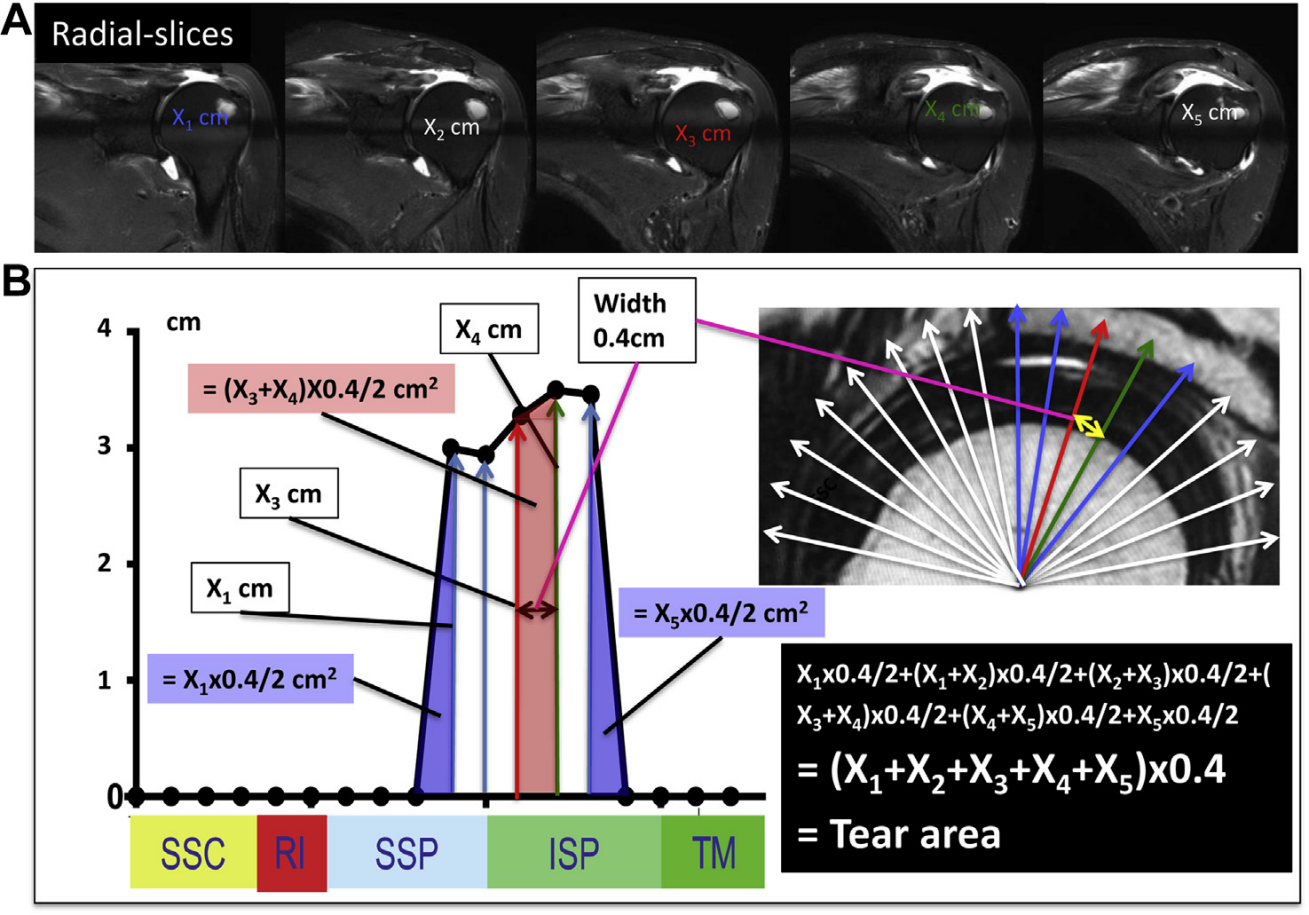
Calculation for the tear area of the rotator cuff. (**A**) The size of the rotator cuff tear was measured for all the slices. (**B**) The formula for the tear area was calculated as the value computed by multiplying the sum of the tear size by the radial slice interval (indicated by *bidirectional arrow*).

The calculation of the tear area of the rotator cuff inluded the following steps: First, the size of the rotator cuff tear was measured in all the slices; second, we defined the tear area as two areas of a triangle and some area of a trapezoid. Thus, the formula for the tear area became easier to obtain and was calculated as the value computed by multiplying the sum of the tear size by the radial-slice interval (0.4 cm) (Fig. 2).

The rotator cuff integrity was evaluated by a postoperative MRI according to Sugaya’s classification during the 2-year follow-up. The intact group was classified as types 1-3. The retear group was classified as types 4-5.^37^

Two shoulder fellowship-trained orthopedic surgeons evaluated the radiographs and MRI scans without information about patient.

### Operative technique and postoperative protocol

All procedures were performed with the patient in the lateral position under general anesthesia. Evaluation of the glenohumeral joint was performed through a posterior portal to confirm the presence of intra-articular injuries such as the degree of the rotator cuff tears and lesions of the biceps tendon. The anterolateral and posterolateral portals were created as working and viewing portals, and a subacromial bursectomy with acromioplasty was performed. After resection of the degenerated tendon stump, the AP and ML lengths of the tear were measured using a calibrated probe under arthroscopic visualization. The intraoperative tear area (cm^2^) was calculated by multiplying the ML and AP length of the tear, as previously described by Wu et al.^41^ Consecutive patients with reparable rotator cuff tear were defined as being able to cover the cartilage surface, including those with a medialization procedure that mobilized the tendon, such that the attachments of the adhesion and coracohumeral ligament were released. If the rotator cuff tear was not reparable, a partial rotator cuff repair or an arthroscopic fascia lata patch graft procedure was performed, and the patient was excluded from this study. The double row suture-bridge technique was used for the rotator cuff repair. Medial row anchors were inserted at the medial edge of the footprint through a small incision made next to the lateral acromial border. QuickPass Suture Lasso (Arthrex, Naples, FL, USA) was used to pass the suture through the intramuscular tendon of the supraspinatus and infraspinatus, and the knots of the medial row anchors were tied. Lateral row anchors were inserted at the lateral side of the greater tuberosity through the anterolateral portal. The number of medial and lateral row anchors depended on the tear size. An immobilizing abduction brace was used for 4 to 6 weeks postoperatively. The rehabilitation program differed according to the tear size. The pendulum and deltoid isometric exercises were initiated on the day after the surgery. During the first 4 to 6 weeks, passive forward flexion exercises were performed. Active exercises were not allowed until 4 to 6 weeks postoperatively.

### Statistical analysis

The chi-square or Fisher’s exact test was used to evaluate the correlation between the nominal and ordinal variables (sex, smoking history, hand dominance, hypertension, hypercholesterolemia, diabetes, and tangent sign) and the presence of retear. Student’s t-test was used to evaluate the correlation between the continuous variables (age; duration of symptoms; BMI; ML and AP tear length; AHI; CSA; fatty infiltration of the supraspinatus, infraspinatus, and subscapularis; occupation ratio; and tear area) and the presence of retear. A multiple logistic regression analysis was performed to identify the independent risk factors. The receiver operating characteristic (ROC) curve was used to obtain the cutoff value of the independent factors and to calculate the area under the ROC curve (AUC). Intraclass correlation coefficients and Cohen k values were used to assess interobserver reproducibility and intraobserver reliability. Agreement by means of intraclass correlation coefficients and k values was rated as excellent for values 0.81-1.0; high, 0.61-0.80; moderate, 0.41-0.60; fair, 0.21-0.40; and poor, ≤0.20. Furthermore, we also investigated the measurement accuracy using Pearson's correlation coefficient to confirm the tear area calculated by radial MRI and the intraoperative tear area.

A *P* value < .05 was considered statistically significant throughout the study. We used a statistical software (SPSS Statistics for Windows, version 25.0; IBM, Armonk, NY, USA) to analyze the data.

## Results

Irreparable rotator cuff tears were seen in 12 patients, and reparable rotator cuff tears in 102 patients. Retears were identified in 15 (14.7%) of the reparable 102 patients on the follow-up MRI scan after the rotator cuff repair. The rates of retears were similar regardless of the age (*P* = .646), dominant hand (*P* = .803), duration of symptoms (*P* = .85), smoking status (*P* = .346), and BMI (*P* = .756). Diabetes mellitus (*P* = .326), dyslipidemia (*P* = .582), and hypertension (*P* = .911) were not significant contributors to retears (Table I).

**Table I:** Preoperative factors.

Factors	Healed group (n = 87)	Retear group (n = 15)	*P* value
Age, y	66.7 ± 0.9	67.8 ± 2.4	.646
Dominant hand	60 (69.0)	11 (73.3)	.803
Duration of symptoms (mo)	9.6 ± 0.7	9.3 ± 2.0	.85
Gender, n (%)			.132
Male	53 (60.9)	9 (60.0)	
Female	34 (39.1)	6 (40.0)	
Smoking status	14 (16.1)	1 (6.7)	.346
Body mass index, kg/m^2^	24.3 ± 0.4	24.7 ± 1.3	.756
Diabetes	14 (16.1)	4 (26.7)	.326
Hyperlipidemia	9 (10.3)	1 (6.7)	.582
Hypertension	21 (24.1)	4 (26.7)	.911

Categorical variables are presented as number (%). Continuous variables are presented as mean ± standard deviation.

In imaging studies, no significant difference was found in the AHI (*P* = .236), CSA (*P* = .927), and fatty infiltration of the subscapularis tendon (*P* = .051) between the two groups. The occurrence of a retear was significantly affected by the AP and ML tear length (*P* < .001, *P* < .001), fatty infiltration of the supraspinatus and infraspinatus (*P* < .001, *P* < .001), occupation ratio (*P* < .001), tangent sign (*P* < .001), and tear area (*P* < .001) as measured using the radial slices (Table II). All these imaging factors except for the fatty infiltration of the subscapularis had excellent intraobserver reliability and interobserver reproducibility (Table III).

**Table II:** Imaging factors.

Factors	Healed group	Retear group	*P* value
Acromiohumeral interval, mm	9.0 ± 0.2	8.3 ± 0.7	.236
Critical shoulder angle, deg	34.1 ± 0.4	34.0 ± 1.3	.927
Anteroposterior tear length by MRI, mm	13.4 ± 0.7	28.2 ± 2.1	<.001
Mediolateral tear length by MRI, mm	14.4 ± 0.9	31.1 ± 2.8	<.001
Occupation ratio of supraspinatus, %	82.8 ± 2.3	62.9 ± 4.5	<.001
Supraspinatus fatty infiltration, n (%)			<.001
Grade 0	10 (11.5)	0 (0)	
Grade 1	50 (57.5)	2 (13.3)	
Grade 2	25 (28.7)	8 (53.3)	
Grade 3	2 (2.3)	5 (33.3)	
Grade 4	0 (0)	0 (0)	
Infraspinatus fatty infiltration, n (%)			<.001
Grade 0	19 (10.3)	0 (0)	
Grade 1	35 (40.2)	4 (26.7)	
Grade 2	31 (35.6)	5 (33.3)	
Grade 3	2 (2.3)	5 (33.3)	
Grade 4	0 (0)	1 (6.7)	
Subscapularis muscle atrophy, n (%)			.051
Grade 0	16 (18.4)	1 (6.7)	
Grade 1	41 (47.1)	5 (33.3)	
Grade 2	28 (32.2)	8 (53.3)	
Grade 3	2 (2.3)	1 (6.7)	
Grade 4	0 (0)	0 (0)	
Tangent sign	6 (6.9)	7 (46.7)	<.001
Tear area by radial MRI, cm^2^	1.8 ± 0.2	10.5 ± 1.3	<.001

*MRI*, magnetic resonance imaging.

Categorical variables are presented as number (%). Continuous variables are presented as mean ± standard deviation. Fatty infiltration was graded according to the criteria by Goutallier et al.^12^

**Table III:** Intraobserver reliability and interobserver reproducibility.

Factors	Intraobserver reliability	Interobserver reproducibility	*P* value
Acromiohumeral interval∗	.835	.871	<.001
Critical shoulder angle∗	.922	.936	<.001
Anteroposterior tear length by MRI∗	.965	.959	<.001
Mediolateral tear length by MRI∗	.985	.971	<.001
Occupation ratio of supraspinatus∗	.942	.912	<.001
Supraspinatus fatty infiltration†	.81	.919	<.001
Infraspinatus fatty infiltration†	.848	.838	<.001
Subscapularis fatty infiltration†	.787	.862	<.001
Tangent sign†	1	.824	<.001
Tear area by radial MRI∗	.989	.993	<.001

∗ Values evaluated with intraclass correlation coefficients.

† Values evaluated with Cohen kappa.

In intraoperative factors, the mean ML length and the mean AP length were significantly longer in the retear group than those in the healed group (*P* < .001). Furthermore, the mean intraoperative tear area that was calculated by multiplying the ML and AP length was larger in the retear group than that in the healed group (*P* < .001) (Table IV).

**Table IV:** Intraoperative factors.

Factors	Healed group	Retear group	*P* value
Subscapularis tear∗	51 (59)	10 (67)	.587
Biceps lesion∗	40 (46)	9 (60)	.405
Mediolateral tear length, mm†	20.8 ± 2.5	35.7 ± 7.5	<.001
Anteroposterior tear length, mm†	16.6 ± 7.5	32.3 ± 12.5	<.001
Tear area, cm^2^†	3.8 ± 2.0	12.1 ± 7.8	<.001

∗ Values expressed as number (%).

† Continuous variables are presented as mean ± standard deviation.

Based on these significant preoperative factors in the univariate analysis, multivariate logistic analyses were performed. The tear area (*P* = .005) was the independent factor that most significantly affected the rate of retear (Table V).

**Table V:** Results of multiple logistic regression analysis.

Factors	Odds ratio	95% Confidence interval	*P* value
Anteroposterior tear length by MRI, mm	0.905	0.727-1.125	.367
Mediolateral tear length by MRI, mm	1.029	0.771-1.373	.846
Occupation ratio of supraspinatus, %	1.107	1.000-1.225	.05
Supraspinatus fatty infiltration	4.946	0.322-75.969	.251
Infraspinatus fatty infiltration	1.088	0.229-5.171	.916
Tangent sign	4.903	0.224-107.301	.313
Tear area by radial MRI, cm^2^	4.876	1.606-14.802	.005

*MRI*, magnetic resonance imaging.

**Figure 3:**
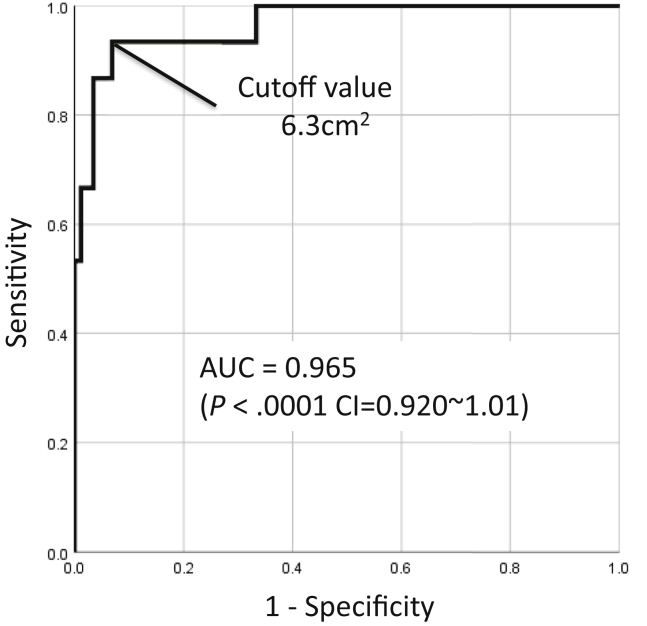
Receiver operating characteristic (ROC) curves for the prediction of retears with the tear area.

ROC curves were plotted only for the significant variables in the multivariate logistic regression analyses. The cutoff values for predicting retears were a tear area of 6.3 cm^2^ with a sensitivity of 86.7%, specificity of 96.6%, and an AUC of 0.965 (Fig. 3). The tear area calculated by radial MRI had a significant correlation with the tear area during the operation (r = 0.8849; *P* < .001).

## Discussion

In this study, the demographic data of the patients, AHI, CSA, tear size, fatty infiltration, muscle atrophy, and tear area, were the factors evaluated to predict the occurrence of retears. This study revealed that the tear area calculated using radial-slice MRI was the only significant risk factor for retear in a multivariate analysis.

Currently, oblique coronal and oblique sagittal MRI scans are used to evaluate the preoperative risk factors for retears, and the tear size, fatty infiltration, and muscle atrophy are considered significant risk factors for retear.

Preoperative fatty infiltration is known to be an important factor affecting the integrity of a repaired rotator cuff. According to Goutallier et al, severe fatty infiltration was correlated with unsatisfactory surgical outcomes and a high retear rate.^13^ High preoperative fatty infiltration grades were significant risk factors for retear.^3^^,^^13^^,^^35^^,^^42^ Especially, the importance of fatty infiltration of the infraspinatus as a prognostic factor was suggested by several studies.^5^^,^^19^^,^^20^ In the study by Jeong et al, only supraspinatus muscle atrophy and infraspinatus fatty infiltration were significant risk factors for retear in the multivariate logistic analyses, and patients with rotator cuff retears had an occupation ratio of the supraspinatus less than 43% or a grade 2 or higher fatty infiltration of the infraspinatus preoperatively (sensitivity, 98.0%; and specificity, 83.6%).^18^ They concluded that muscle quality is the most important factor associated with retear. In contrast, some reports have shown that fatty infiltration and muscle atrophy were not independent predictors of postoperative rotator cuff integrity.^2^^,^^11^^,^^36^ Gladstone et al reported that fatty infiltration emerged as an independent predictor of outcome, apparently overriding the effects of tear size or repair integrity.^11^ This study reported that there were no statistically significant differences in the degrees of fatty infiltration of the supraspinatus and infraspinatus in the multivariate analysis. The tear area and fatty infiltration of the rotator cuff may be significantly correlated.

Le et al reported that the intraoperative tear size and tear area were significant predictors of the retear rate.^22^ In addition, Wu et al reported that the tear area was the best intraoperative predictor of repair integrity after rotator cuff repair, with tears less than 2 cm^2^ twice as likely to heal than tears greater than 6 cm^2^.^41^ Therefore, the tear area is strongly associated with postoperative rotator cuff integrity. However, the method of measuring the intraoperative tear area listed in these studies was a value obtained by simply multiplying ML and AP tear length, and it is considered that the same value may be obtained even if the tear morphology is different and were assessed using the predictive model of intraoperative factors. In contrast, we developed a method to measure the tear area using radial-slice MRI preoperatively. The ability to predict retear preoperatively is of great benefit to surgeons and patients in terms of treatment choices and prediction of postoperative outcomes.

Radial-slice MRI was applied to visualize the acetabular labrum of the hip.^15^^,^^16^ Munk et al first described examination of the glenoid labrum of the shoulder joint in 1989.^30^ Radial-slice MRI centered on the humeral head provide a cross-slice perpendicular to the insertion in all slices of rotator cuff. However, crosstalk artifact caused impractical quality of labrum and rotator cuff and was not clinically applied. In recent year, remarkable advances in MRI overcame disadvantages of radial-slice MRI. Furukawa et al^10^ reported that radial-slice MRI was useful for diagnosing subscapularis tendon tear by achieving a reduction in the partial-volume effect and potentially offering clear visualization of the subscapularis tendon. They suggested that because radial-slice MRI was capable of imaging sections perpendicular to the anterosuperior and posterosuperior tendon insertion.

Therefore, radial-slice MRI makes it possible to measure the size of the rotator cuff tear specifically associated with infraspinatus tendon or subscapularis tendon by reducing the partial-volume effect and to perform a quantitative assessment calculated by the formula for the tear area. Furthermore, this study showed that the tear area was the only significant risk factor for rotator cuff retear; we also concluded that the retear risk was very high in cases where the tear area was more than 6.3 cm^2^. The Goutallier classification is a semi-quantitative evaluation. In cases with fatty degeneration and rotator cuff tears, it may be difficult to determine whether the rotator cuff muscle is Goutallier grade 2 or Goutallier grade 3.^7^ In addition, Meyer et al showed the retear rates for Goutallier grade 1, 2, and 3 were 43%, 67%, and 100%, respectively.^27^ From these results, it is difficult to judge repair is possible only by Goutallier grade. On the other hand, imaging evaluation of the tear area is a reliable quantitative method and critical in clinical decision-making for rotator cuff tears. In addition, a tear size of 6.3 cm^2^ was the strongest predictor of retear with an AUC of 0.965, sensitivity of 86.7%, and specificity of 96.6%. The results of the AUC were considered excellent for AUC values between 0.9 and 1, good for AUC values between 0.8 and 0.9, fair for AUC values between 0.7 and 0.8, poor for AUC values between 0.6 and 0.7, and failed for AUC values between 0.5 and 0.6.^26^ These results indicated that our evaluation method was a viable alternative to the Goutallier classification system for assessing the preoperative risk factors for retear, and if the preoperative tear area is 6.3 cm^2^ or more, even if it looks reparable during the operation, there is a high possibility that retear will occur after the operation, so it is better to perform superior capsule reconstruction.

In addition, radial-slice MRI imaging method is the same as the conventional setting MRI imaging technique except for the slice setting. Imaging acquisition time is approximately the same as that of conventional slices, about four minutes. Moreover, because it is possible to accurately visualize all the rotator cuff attachments orthogonal to each part of the head of the humerus with radial-slice images, it is considered that the oblique coronal and axial images can be covered. Therefore, diagnosis of rotator cuff tear, including supraspinatus tendon, infraspinatus tendon, and subscapularis tendon, may be possible only with radial slices and oblique sagittal slices.

This study has several limitations. First, the retrospective design made the study highly dependent on the data obtained from the interpretation of MRI scans. Second, the rehabilitation program differed based on the tear size. Third, we did not focus on the postoperative factors. Fourth, we did not include shoulder activity and physical examination results as patient risk factors. Fifth, all surgical procedures had been performed by a single surgeon. Finally, we did not evaluate associations between the tear area and postoperative functional outcomes. Nevertheless, this study is, to our knowledge, the first to evaluate preoperative risk factors for retears using radial-slice MRI and the first to show a significant association between the tear area and postoperative rotator cuff integrity. Furthermore, the tear area had accurate cutoff values of 6.3 cm^2^, respectively. From these results, if the tear area exceeds 6.3 cm^2^, even if it looks repairable during the operation, it may be better to perform alternative surgery without primary repair.

## Conclusion

This study concluded that the tear area calculated using radial-slice MRI was a preoperative risk factor for retears of the rotator cuff. The tear area was the independent factor that most significantly affected the rate of retear and showed excellent accuracy with a cutoff value of 6.3 cm^2^. Radial-slice MRI may be helpful as a diagnostic tool for accurately assessing the postoperative rotator cuff integrity and suggesting adequate treatment measures.

## Disclaimers

Funding: No funding was disclosed by the authors.

Conflicts of interest: The authors, their immediate families, and any research foundations with which they are affiliated have not received any financial payments or other benefits from any commercial entity related to the subject of this article.
